# VOADS AND OLDER ADULTS IN DISASTERS

**DOI:** 10.1093/geroni/igz038.1439

**Published:** 2019-11-08

**Authors:** Mary H mcsweeney-feld

**Affiliations:** towson university, Towson, Maryland, United States

## Abstract

Voluntary Organizations Active in Disasters (VOADs) are a key mechanism for sharing knowledge and resources throughout a disaster cycle. VOADs work as a coalition of not-for-profit organizations that can respond to disasters on a local level in a coordinated response. Given the changes in FEMA’s Strategic Plan for 2018-2022, the role of VOADs will become more important over time. It is unclear whether the needs of older adults in disasters will be addressed by VOAD coalitions. Organizations such as public health departments and emergency management services are typically involved in the activities of VOAD organizations, but it is unclear whether organizations serving older adults know about VOAD coalitions or have considered becoming members of these organizations. A case study of the state of Maryland’s VOAD coalition will be used as an example of how one state VOAD is able to provide services and supports for older adults in disasters.

